# Climate change will decrease the range size of snake species under negligible protection in the Brazilian Atlantic Forest hotspot

**DOI:** 10.1038/s41598-019-44732-z

**Published:** 2019-06-12

**Authors:** Ricardo Lourenço-de-Moraes, Fernando Miranda Lansac-Toha, Leilane Talita Fatoreto Schwind, Rodrigo Leite Arrieira, Rafael Rogério Rosa, Levi Carina Terribile, Priscila Lemes, Thiago Fernando Rangel, José Alexandre Felizola Diniz-Filho, Rogério Pereira Bastos, Dayani Bailly

**Affiliations:** 1 Programa de Pós-graduação em Ecologia de Ambientes Aquáticos Continentais (PEA), Universidade Estadual de Maringá, Maringá, Paraná, Brazil; 2 Laboratório de Herpetologia e Comportamento Animal, Universidade Federal de Goiás, Goiânia, Brazil; 3 Programa de Pós-Graduação em Biologia Comparada (PGB), Universidade Estadual de Maringá, Maringá, Paraná Brazil; 4 Laboratório de Macroecologia, Universidade Federal de Goiás, Regional de Jataí, Jataí, Goiás Brazil; 5 Departamento de Zoologia, Instituto de Biociências, Universidade Estadual Paulista Júlio de Mesquita Filho (UNESP), Rio Claro, São Paulo Brazil; 6 Departamento de Ecologia, Instituto de Ciências Biológicas, Universidade Federal de Goiás, Goiânia, Goiás Brazil

**Keywords:** Climate-change ecology, Projection and prediction

## Abstract

Reptiles are highly susceptible to climate change, responding negatively to thermal and rainfall alterations mainly in relation to their reproductive processes. Based on that, we evaluated the effects of climate change on climatically suitable areas for the occurrence of snakes in the Atlantic Forest hotspot, considering the responses of distinct reproductive groups (oviparous and viviparous). We assessed the species richness and turnover patterns affected by climate change and projected the threat status of each snake species at the end of the century. We also evaluated the effectiveness of the protected areas in safeguarding the species by estimating the mean percentage overlap between snake species distribution and protected areas (PAs) network and by assessing whether such areas will gain or lose species under climate change. Our results showed greater species richness in the eastern-central portion of the Atlantic Forest at present. In general, we evidenced a drastic range contraction of the snake species under climate change. Temporal turnover tends to be high in the western and north-eastern edges of the biome, particularly for oviparous species. Our predictions indicate that 73.6% of oviparous species and 67.6% of viviparous species could lose at least half of their original range by 2080. We also found that existing protected areas of the Atlantic Forest Hotspot have a very limited capacity to safeguard snakes at the current time, maintaining the precarious protection in the future, with the majority of them predicted to lose species at the end of this century. Although oviparous and viviparous snakes have been designated to be dramatically impacted, our study suggests a greater fragility of the former in the face of climate change. We advocated that the creation of new protected areas and/or the redesign of the existing network to harbour regions that maximize the snake species occupancy in the face of future warming scenarios are crucial measures for the conservation of this group.

## Introduction

Climate change is a prevalent threat to global biodiversity^1,2^. The potential changes in physiological and ecological processes promoted by climate change may affect the distribution and persistence of species in an environment^3,4^. The complex relationship of this changing process with organisms has been addressed from various perspectives, with studies evaluating how climate changes affect the individual performance^5,6^, demographic dynamics^7,8^, species composition and species richness^9,10^. Predicted outcomes include adaptation to novel conditions^11^, shift, expansion or retraction of ranges^10,12^, isolation to unaffected areas or climatic refuges^13–15^ and extinction events^1^. There is a growing consensus that management decisions for biodiversity conservation must be taken in light of the potentially catastrophic effects of climate change^16^.

Protected areas (PAs) typically figure as the cornerstone of conservation strategies, covering about 14.7% of the terrestrial Earth’s surface^17,18^. The effectiveness of such areas in achieving conservation goals depends partly on how well represented the ecological diversity is in a network of designated lands^19^. In this sense, PAs may be particularly helpful in safeguarding the global biodiversity in the face of habitat loss^20,21^. However, as they confront climate change, such areas could lose the effectiveness of their conservation function, mainly due to changes in species ranges. This trend is alarming from a conservationist’s viewpoint, because if these areas fail to protect species in the future, the current biodiversity crisis would reach unprecedented levels^12^. Thus, there is an emerging need for conservation plans to abandon their traditionally static nature and move toward a more dynamic conservation criterion that takes the climate instability into account^22^. For now, most solutions offered by conservation scientists and practitioners to deal with the effectiveness of conservation units in the future involves the establishment of new PAs harbouring areas with future suitable climate^9,23,24^.

Ecological niche models (ENMs), also referred to as species distribution models (SDMs)^25,26^, have been increasingly used to estimate species ranges under future scenarios of climate change. Versions of these models employ different statistical and computational methods, ranging from simple processes of the construction of bioclimatic rules to complex artificial intelligence techniques^26,27^. Geographical projections of ranges arising from ENMs’ outputs can be overlaid onto a layer of PAs to assess the areas expected to retain suitable climatic conditions for different species^24,28,29^. This evaluation is useful to indicate the areas with maximum match to current climate or to point out new areas of future conservation value^9^, and to evaluate the current and future effectiveness of PAs in harbouring species^10,12,30^.

Ectothermic animals are highly susceptible to climate change^31,32^ due to the interdependence of their behavioural-physiological functions in relation to the external environment^33,34^. Reptiles, in particular, respond negatively to both thermal and rainfall changes, mainly in relation to their reproductive processes^4,35^. In snakes, temperature and humidity affect the species differently, according to their reproductive mode^35,36^. Reproductive rates in oviparous species are negatively impacted by the increase in temperature and decrease in rainfall, considering that their nests lose the required moisture for embryonic development of eggs^36,37^. In viviparous species, the increase in body temperature can promote drastic energetic costs during the stages of vitellogenesis, gestation, parturition, and post-parturition recovery, which can cause the death of females and their offspring^38,39^. Although changes in temperature and rainfall tend to negatively affect the physiological processes of both reproductive groups, viviparity provides important advantages because mothers are able to reduce the effects of environmental restrictions through careful behavioural thermoregulation^40,41^. Thus, reproduction could to be a crucial factor determining the capacity of snake species to colonize different habitats, influencing geographical distribution processes^39^. In this context, it’s important to assess if snake species with these two reproductive modes tend to respond differently to climate change with respect to their geographical distribution.

In our study, we assess the effects of climate change on climatically suitable areas for the occurrence of snake species in the Atlantic Forest, a biodiversity hotspot for conservation^42^. This biome, which is marked by rich diversity of endemic species, have been largely impacted by poor soil management, extensive destruction and simplification of the forests, and hence fragmentation^42,43^. Based on ecological niche modelling, our study relies on the correlations between climate and snake species’ occurrences to estimate current and future richness patterns, as well as turnover tendencies. Considering that snakes of distinct reproductive groups respond in a particular way to alterations in climate conditions^36–41^, they may also respond differently with respect to their geographical distribution given their habitat requirements. Based on this premise, we deconstructed the responses of the snakes into two functional reproductive modes (oviparous and viviparous) to identify process underlying the general distributional pattern. By predicting the current and future range size of species, we also explore, in a descriptive sense, the threat status of snake species projected at the end of the century while affected by climate change. Finally, we evaluate whether current locations of PAs in the Atlantic Forest Hotspot are efficient in protecting snake diversity in the face of climate-induced shifts in species richness inside PAs.

Due to the ectothermic features of snakes, an analysis with ecological niche models can reflect more accurately the possible direction of species facing the climatic changes. Furthermore, snakes are pointed out as susceptible to climate change partially due to their low population size and poor dispersal capacity^44,45^. Nevertheless, studies assessing the potential impacts of climate change on snakes’ distribution in Brazil, and specifically in the Atlantic Forest Hotspot are lacking. Besides that, most research and conservation efforts have prioritized iconic species with socio-economic interest^46^ and snakes have been looked down upon due to negative public and social perceptions^47^. By introducing a multi-species analysis within the climate change context, identifying the species that require more conservation attention as well as analysing the effectiveness of PAs in safeguard species, our study provides useful information that aggregated to findings of other taxa should help to improve conservation strategies in the Atlantic Forest Hotspot.

## Results

The first two PCA axes explained a large proportion of variation between species richness maps generated by different ENMs. The accumulated proportion of variation explained by the two axes ranged from 87.3% to 93.3% for viviparous species in 2080 and oviparous species in the current time, respectively (see Supplementary Table S1). In general, BIOC and GOWD produced similar results in both time periods for both reproductive groups (see Supplementary Fig. S1). The predictions of MAXENT were similar to ENFA, especially for oviparous species. The CONS model had the highest loading for the first PCA axis, reflecting the main direction of variation among suitability maps (Supplementary Table S1). Consequently, only the outputs derived from the consensus of multiple ENMs (CONS) were retained for interpretation.

**Figure 1:**
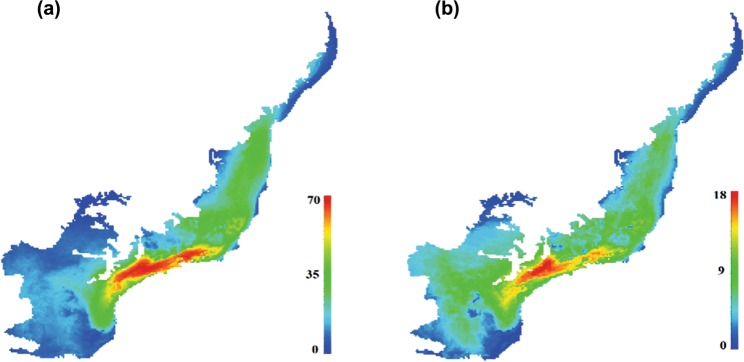
Species richness of snakes distributed in the Atlantic Forest in the current time. Consensus map derived from ensemble of the ecological niche models (BIOC, GOWD, MAXE and ENFA) showing the species richness patters for oviparous (**a**) and viviparous (**b**) species in the current time.

The overlap of individual species ranges generated by the CONS model provided evidence that highest species richness values were restricted to the eastern-central portion of the hotspot in the current time (Fig. 1). Future predictions produced by CONS from different AOGCMs pointed out losses of climatically suitable areas in this region by 2080 for both reproductive groups.

**Figure 2:**
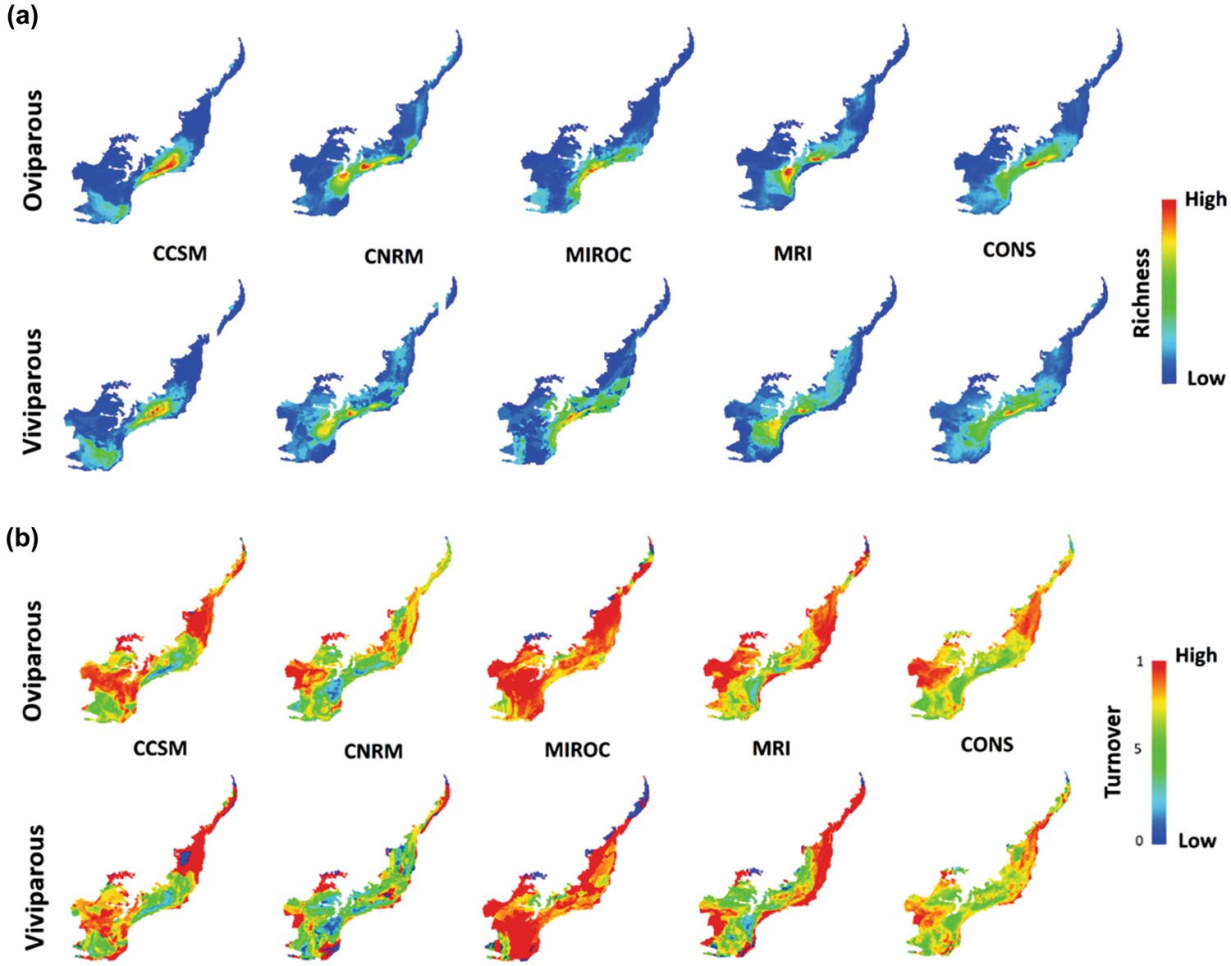
Species richness and turnover patters of snake species across Atlantic Forest Hotspot in the future time. Species richness (**a**) and turnover (**b**) derived from different Global Circulation Models (CCSM, CNRM, MIROC and MRI) and consensus model (CONS) for oviparous and viviparous species in the future time (2080).

In general, CNRM and MRI showed two distinct species-rich areas, whereas CCSM and MIROC produced more homogeneous results, with the latter being more restrictive (Fig. 2a). The full ensemble model showed that in the future the species richness peaks will be restricted to a reduced portion of the central-eastern region of the biome, in locations closer to the mountains of the Atlantic Forest (Fig. 2a, last column). Similar to species richness, the turnover outputs were distinct between GCMs (Fig. 2b). In general, CCSM and MRI produced similar results for both reproductive groups. Already CNRM presented lower turnover values and MIROC overestimated the composition changes across the biome. By combining the results of AOGCMs in a full ensemble model, we observed that the temporal turnover was high, especially for oviparous species (Fig. 2b, last column). Changes in the species composition are predicted to be greater on the western edge and on the north-eastern edge of the biome, especially for oviparous species. Values above 0.8 were found in 45.0% and 32.5% of cells covering Atlantic Forest territory for oviparous and viviparous species, respectively.

**Figure 3:**
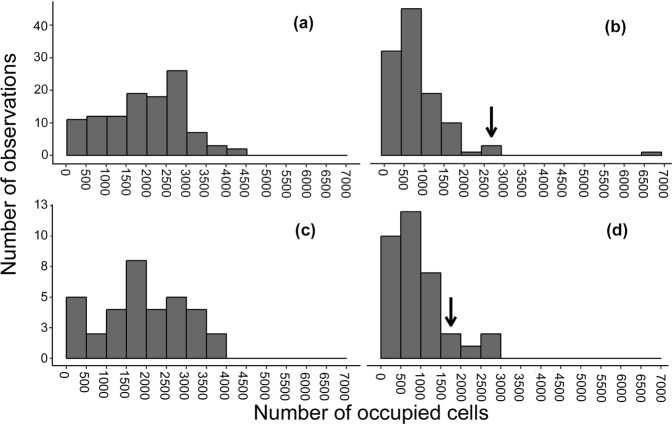
Expected changes in the range size of the snakes in the Atlantic Forest Hotspot due to the future climate effects. Range size for oviparous species in the current time (**a**) and 2080 (**b**) and for viviparous species in the current time (**c**) and 2080 (**d**). Arrows indicate the size class harbouring the mode of observations in the histogram of the current time.

We observed that oviparous species presented a broader distribution than viviparous species in the Atlantic Forest in current time, with the most species occurring in between 2,500 and 3,000 cells (Fig. 3a). Currently, most viviparous snakes occupy between 1,500 and 2,000 cells (Fig. 3c). Our results illustrated a drastic range contraction of snakes in the Atlantic Forest Hotspot under climate change, especially for oviparous species, for which the mode of observations was reduced in four size classes (Fig. 3b). For viviparous species, the mode of observations was reduced in two size classes (Fig. 3d). For both reproductive modes the majority of species should occupy only between 500 and 1,000 cells in the future (Fig. 3b,d). Nonetheless, it was possible to observe that one oviparous species tends to greatly expand its geographic range with climate change, occupying between 6,000 and 7,000 grid cells in future (Fig. 3b; see details below).

**Figure 4:**
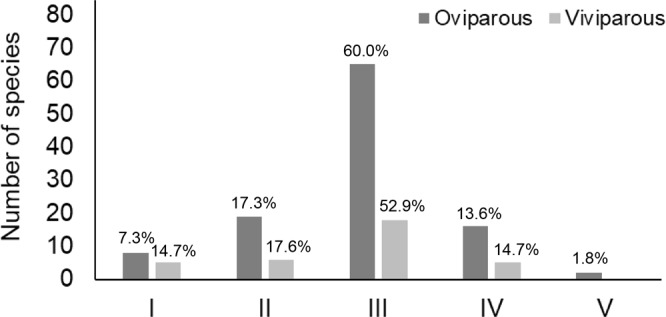
Range loss projected to 2080. I. species whose loss is estimated at <30% for the projected time interval; II. species whose loss is estimated at 30% for the projected time interval; III. species whose loss is estimated at 50% for the projected time interval; IV: species whose loss is estimated at 80% for the projected time interval and V. species whose loss is estimated at 100% for the projected time interval.

Our results indicated that under the high loss of habitat suitability, 73.6% and 67.6% of oviparous and viviparous species, respectively, should lose at least 50% of its original range by 2080 (Fig. 4). Only 7.3% of oviparous species and 14.7% of viviparous species should lose less than 30% from the original distribution area of the species, given the restrictions imposed by future climate conditions. According to IUCN criterion B1, 15.45% of oviparous species and 11.76% of viviparous species are projected to be threatened by 2080 (Supplementary Table S2). Our results also indicated that the oviparous species *Phalotris lemniscatus* and *Clelia hussami* are expected to be extinct from the biome by 2080. Conversely, two oviparous species were projected to benefit with climate change; *Xenodon merremii* should increase 37% of its range and *Drymarchon corais* should increase 14% of its range. The oviparous species *Chironius carinatus* was the only species practically unaffected (habitat losses of only 0.2%). The summary of the impacts of future climatic changes on each individual species is given in Supplementary Table S2.

**Figure 5:**
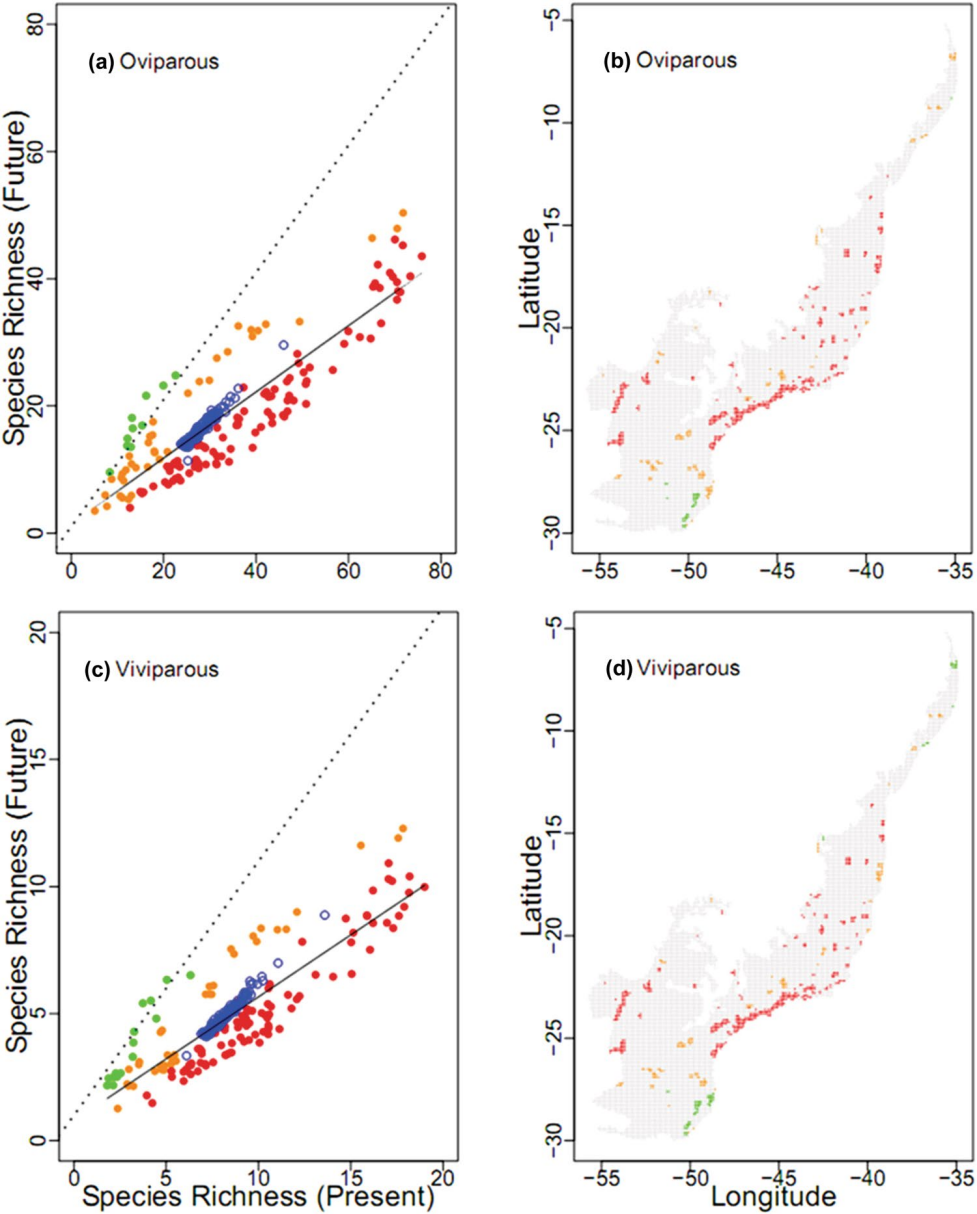
Effectiveness of the protected areas of Atlantic Forest in safeguarding snake species. Relationship between present and future snake species richness, (**a**) for oviparous species and (**c**) for viviparous species. Open blue circles correspond to richness according to a null model of random location of protected areas in the biome. Filled red circles indicate protected areas predicted to have severe species richness losses. Orange filled circles indicate protected areas predicted to have mild species richness losses. Green filled circles indicate protected areas predicted to gain species richness. Dashed lines are the extrapolated regressions of the expected species richness according to the null model. Solid lines indicate the regression of modelled species richness in the future against modelled species richness in the present. Maps of protected areas predicted to gain (green) or lose (orange, red) species in future changing (**b**) for oviparous species and (**d**) for viviparous species.

The comparison provided by the null model indicating the gains and losses of species in PAs in the face of climate change revealed great variation between how many snake species the PAs would lose (and also gain in case of distance methods). For the vast majority of real locations of PAs the average richness for current and future climatic scenarios was lower (red dots in Fig. 5a,c) than those obtained from the random locations of PAs (blue dots in Fig. 5a,c). Thus, for both reproductive groups the most PAs should become highly climatically unsuitable, suffering severe species richness losses (red protected areas in Fig. 5b,d), especially along the coast or in adjacent mountains. The number of PAs predicted to experience mild species losses under climate change was also high (orange protected areas, Fig. 5b,d). Conversely, the number of PAs predicted to gain species (green dots in Fig. 5a,c) was very low. Areas with such characteristic are located in the cooler southern region of the Atlantic Forest for both reproductive modes (green protected areas in Fig. 5b,d), and in the extreme northern portion of biome for viviparous species (green protected areas in Fig. 5d).

**Figure 6:**
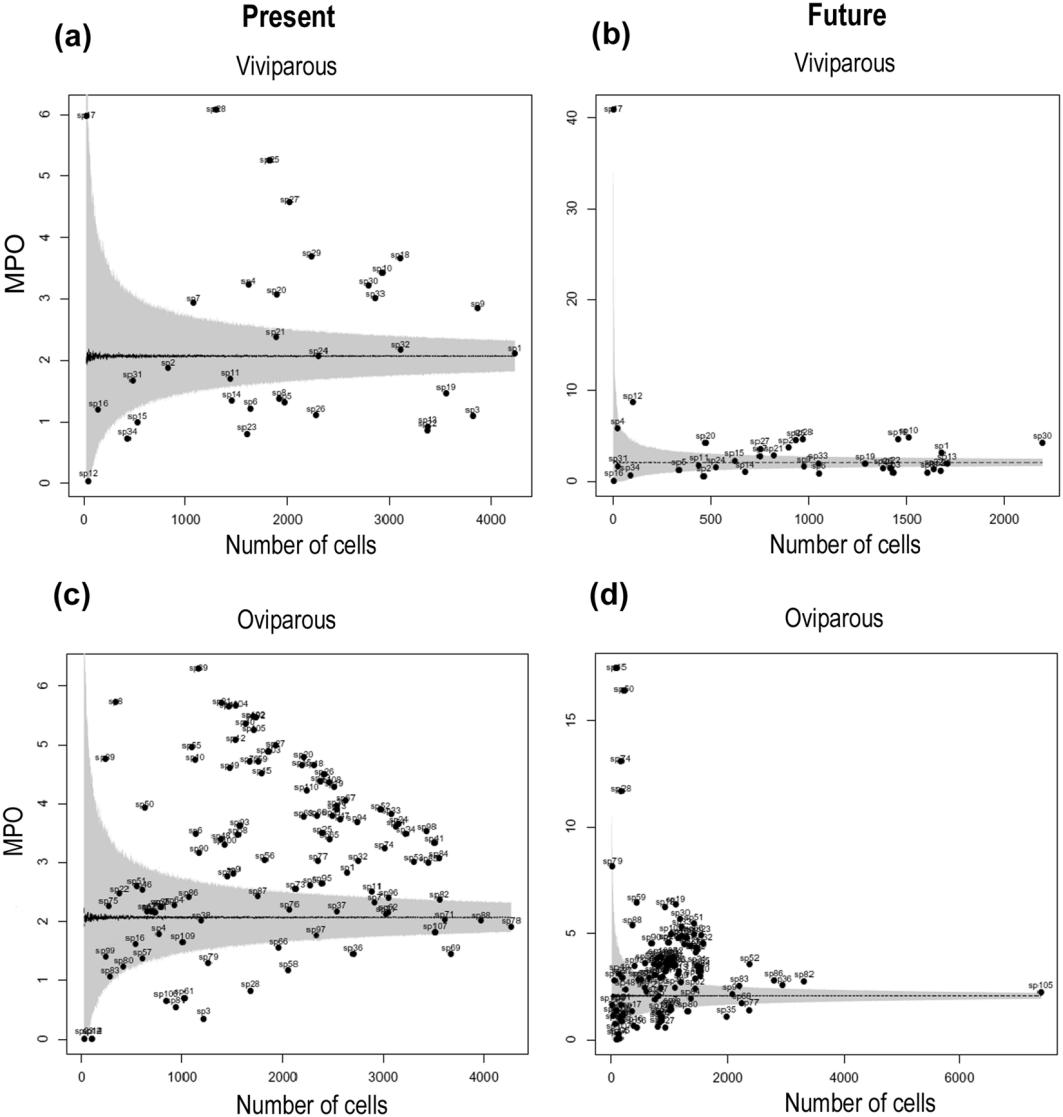
Relationship of the mean percentage overlap (MPO, percentage of spatial overlap between snake species distribution and protected areas network in Atlantic Forest Hotspot) and the number of cells occupied by the species at present and future (2080). Species are numbered and represented by black dots. The dashed lines show mean percentage overlap from 1,000 randomisations, grey surface represents the random range, which indicates the range of the 95th percentiles of the randomised data; (**a**,**b**) indicate MPO relationship for viviparous snake species at present and future times respectively; (**c**,**d**) indicate MPO values of oviparous snake species for present and future respectively.

The analysis of the mean percentage overlap (MPO) between snake species distribution and protected areas (PAs) network in Atlantic Forest revealed a negligible protection of both reproductive groups. At the current time, the mean percentage overlap was 2.33% (ranging from 0.03 to 6.08%, SD = 1.51%) for viviparous (Fig. 6a and Table S3) and 3.06% (ranging from 0.00 to 6.29%, SD = 1.44%) for oviparous species (Fig. 6c and Table S4). At the future time, the mean percentage overlap was 3.63% (ranging from 0.00 to 40.91, SD = 6.83%) for viviparous (Fig. 6b and Table S3) and 3.36% (ranging from 0.00 to17.48%, SD = 2.75%) for oviparous species (Fig. 6d and Table S4). The MPO relationship exhibited a polygonal form, evidencing a more precarious conservation scenario for the species located inside, and mainly below of the random range, for which the protection from existing PAs is respectively equal to or worse than the expected by a random distribution of the species (Fig. 6). Within the scenario of scant protection of snakes, 35.29% and 32.35% of the viviparous species presented MPO higher than expected by chance at present and future times, respectively, and 38.23% and 29.41% of the species presented MPO not significantly different than that expected by chance at present and future times, respectively (Fig. 6a,b and Table S3). More importantly, the representativeness was lower than expected by chance for 32.35% and 29.41% viviparous species at present and future times, respectively (Fig. 6a,b and Table S3). For the oviparous snakes, we found that 60.00% and 57.40% of the species presented level of representativeness in PAs higher than expected by chance at present and future times, respectively. For 28.18% and 26.85% of the oviparous species, the MPO values were not significantly different than expected by chance at present and future times, respectively (Fig. 6c,d and Table S4). In even less favourable conditions are 11.81% and 15.74% of the oviparous species, for which the MPO values were lower than expected by chance at present and future times, respectively (Fig. 6c,d and Table S4).

## Discussion

Our study showed that the changes and losses of climatically suitable areas in the Atlantic Forest projected for 2080 can trigger important range shifts of snake species, with most species suffering range contractions towards their distribution centers. In these cases, the persistence of species in their original ranges will be dependent on the degree of physiological and phenotypic plasticity or evolutionary adaptation of each species to changing conditions (either abiotic or biotic), or a combination of these processes^48–50^. Nonetheless, the behavioural plasticity of snakes to adjust to suboptimal thermal conditions seems to be considerably limited^51^. In addition, reptiles in tropical forests have less thermoregulation options due to the relative thermal homogeneity of these habitats^52,53^. Thus, tropical reptiles become sensitive to climate change due to their narrow thermal tolerances, which gives the group a reduced thermal safety margin^4^. In addition, due to the large increase in their metabolic rates in response to global warming, tropical reptiles should experience the increased need for food and an increased vulnerability to starvation, leading possibly to a reduction in discretionary energy for reproduction^54^.

However, besides the thermoregulation and tolerance of the species, structural features of habitats can mitigate the effects of climate change addressed here. When the thermal conditions increase, abundantly shaded environments can provide favourable conditions and serve as thermal refuges or retreat sites^55,56^. The original features of the Atlantic Forest could efficiently provide such a subsidy, but human activities have destroyed about 88.3% of the original vegetation^43^, thus restricting the retreat sites to only 11.7% of Atlantic Forest, highlighting the east-central region, where the last great remaining forests remain^43,57^. This more pristine condition of the habitat could explain the greater species richness in this region for reproductive groups, as well as lower losses and turnover of species. Also, it is expected that the retraction of ranges predicted for 2080 reduce species richness peaks for the very small portion of this region that is close to mountains. Species with access to mountainous regions may migrate to higher altitudes, which have lower temperatures^58^ and in the case of the Atlantic Forest, should retain greater humidity by well-preserved forest cover.

Our results showed that mainly the southwest and northeast regions of the Atlantic Forest should experience great losses of habitat suitability and a greater turnover of snake species over time. These areas have been severely impacted by human activities. Forests in the northeast have been largely devastated by cattle ranching and the timber industry^59^, while large-scale sugarcane farming and cattle ranching are widely spread throughout the biome’s southwest region^60,61^. Due to growing biofuel demands, the sugarcane farming is expected to increase in Brazil as a whole, replacing livestock and cattle farming^62^. These changes could bring about potential conservation conflicts for these particular regions of the Atlantic Forest.

Our data indicate that Atlantic Forest snakes, especially oviparous species, may suffer drastic range loss by 2080. Among the more alarming results is the predicted extinction of *Phalotris lemniscatus* and *Clelia hussami* from the biome. The former is a species distributed from the state of Santa Catarina in Brazil to Uruguay^63^ and the latter is an endemic species of the Atlantic Forest^64^, for which habitat suitability losses will imply global extinction. In addition, *P*. *lemniscatus* has only 1.23% of its current distribution covered by the existing PAs, and *C*. *hussami* has its current distribution area completely unprotected (MPO = 0.00%; see Table S4), which tend to decisively contribute to the disappearance of these species from the biome irrespective of climate change effect, since unprotected ranges expose the species to multiple, impactful human stressors. Conversely, *Xenodon merremii* and *Drymarchon corais* should benefit from climate change. These oviparous species have opportunistic dietary habits and a wide geographic distribution, and can also be found in open areas in the Cerrado and Caatinga biomes^65,66^ demonstrating physiological plasticity with respect to different climates.

Although to a lesser extent, our results show that viviparous species should also suffer a great impact by a changing climate, with 67.6% of species predicted to lose at least half of their current distribution. The endemic viviparous species *Bothrops pirajai* and *Corallus cropannii*^64,67^ which have been currently evaluated as endangered and vulnerable, respectively, on the national threatened species list, were projected to both be endangered at the end of this century as a result of the climate change. It should be noted that viviparous species confer an evolutionary advantage by allowing females to keep embryo temperature constant, which it would be a benefit mainly in climatically unstable regions^41,68^. However, it is important to mention that viviparity is demanding in energetic terms and affects the capacity of females to feed, disperse, and escape from predators^69^. In addition, the current fragmentation of forests due to the urbanization and agricultural frontier expansion may prevent the dispersal of species^59–62^.

It is noteworthy that among the 144 snakes evaluated in this study, 80.0% of oviparous species and 73.5% of viviparous species are not evaluated by the IUCN^70^ and 99.1% of oviparous species and 94.1% of viviparous species are not evaluated by the Brazilian Red List. From a conservation perspective, snakes have been neglected^71^, attracting fewer conservation efforts when compared to the “flagship” organisms, such as birds and mammals. Consequently, only highly endangered species have benefited from practical conservation programs^72,73^. Nonetheless, there is evidence that terrestrial snake populations have undergone declines around the world, even in well-protected locations^74^, which is consistent with our results.

Effects of climate change will drive organisms (including reptiles) to extinction, but the magnitude of the effects depend on whether species are able to alter their geographic distribution in response to climate change^75^, especially for snake species^45,76^. Species’ range shifts, however, should move species out of PAs^77^ and local extinction could change the species composition within these protected sites^78^. Thus, the effectiveness of PAs will depend on the ability to maintain native species in their habitats, ensuring conservation in the long-term^30,79^, since conserving snakes in anthropogenic environments can be problematic^80^. In this sense, our study points out a very limited capacity of the existing PAs to protect snakes at the current time and in the face of climate change. The mean percentage of overlap between snake species distribution and PAs territories ranged from 2.63% to 3.63% considering both reproductive groups and times. These are values markedly lower than those reported for reptiles, amphibians and bats from Europe^81,82^. The poor performance of Brazilian PAs in protecting a snake species was also evidenced in the Cerrado and Caatinga biomes^24^.

**Figure 7:**
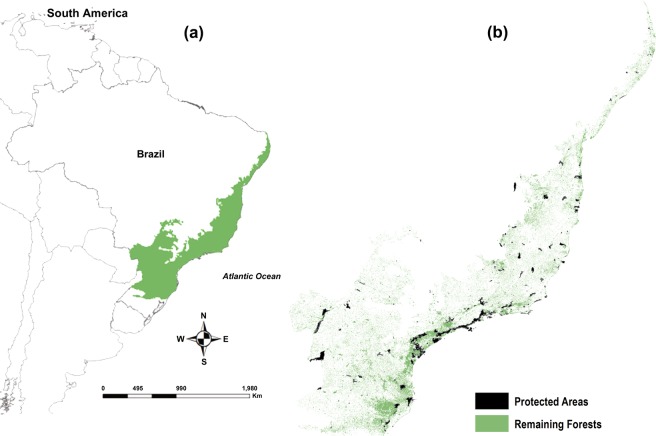
Brazilian Atlantic Forest Biodiversity Hotspot. Original extent of the Atlantic Forest (in green) in South American territory (**a**). Location of protected areas (in black) in the Atlantic Forest (**b**).

Regardless of the reproductive group, the conservation scenario is especially worrisome for the snake species in the lower left corner of the MPO relationship polygon, which have restricted distributions and practically no protection from PAs network (see Fig. 7). Among the more alarming cases are *Chironius carinatus*, which is entirely unprotected by existing PAs at current and future times and *Bothrops pirajai*, which has less than 2% protection at current time and completely loses protection at future time. *Clelia hussami*, which is not currently protected by the existing PAs tends to become extinct in the future due to the absence of suitable environmental-climatic conditions. In this sense, we highlight the role of our study in contributing to the adoption of focal management measures to long-term protection of the Atlantic Forest snakes most prone to extinction in the face of projected climate change.

In addition, our analyses pointed out that the major negative impact of climatic changes should occur along the PAs in the coastal line of the biome. It is also important to mention that the effectiveness of the few PAs in the south (i.e., National Park of Serra Geral) and extreme north (i.e., Biological Reserve Saltinho), here demonstrated by the gain of species in the future, is dependent on the capacity of the snakes to reach such areas. Snake species have a very low vagility^83,84^. Thus, it is possible that climatically suitable PAs will not receive new snake species in the future due to the intrinsic dispersal limitation of this group, and by the lack of essential biotic interactions^69,85^. Future conservation efforts should be fully aware that the distribution of snake species may become altered and that species extinction is a possible outcome.

Different ENM methods yield different predictions which are consistent with results from different comparative studies^86–88^. According to studies suggesting that better predictions can be achieved through the consensus of multiple models^89,90^, our consensus model showed more consistent results for both reproductive groups, reflecting the main direction of variation among species richness maps. However, when multiple species are considered, the choice of how the individual species will be treated generally should have more impact on outcomes than decisions regarding the choice of the analytical algorithms^91,92^. In our study, the range of each individual species was previously modelled and then overlapped to obtain richness maps. This approach enables each species to respond in an individualistic manner to the environmental gradient^91^, instead of other methods that assume the unrealistic condition that the functional form of these responses is the same across all species^91,93^. Thus, the approach used in this study generates more detailed richness and turnover maps discretely bound by the limits of each individual species. However, it is important to mention that ENM predictions are subject to uncertainties inherent to the modelling process per se^92,93^. For instance, uncertainties from spatial coverage of samplings, characteristics of training data, decision thresholds, variables selection, reliability of absence data and presence-only models challenge the production of accurate species distribution outputs^94–96^. In our study, we used presence-only models to predict the impacts of the climate change on the snakes of the Atlantic Forest Hotspot. Although presence-only models can embed some variability in the predictions, they have been the only alternative when absence data are unavailable or impossible to obtain^97–99^, a common scenario for many taxa, especially for those of the megadiverse tropics, as is the case of the Atlantic Forest snakes. Nonetheless, we highlight that ensemble forecast approach has an important role in minimizing uncertainties and errors from ENMs^92^.

Though the difference in responses of species belonging to two reproductive groups is expected to be tenuous due to phylogenetic similarity, we concluded that the greater temporal dissimilarity of species composition and the more drastic range contractions of oviparous species are indicative of a greater frailty of these species in the face of climate change in terms of habitat requirements. Considering the physiological and behavioural particularities of both reproductive modes reported by the literature, it is possible that, in addition to the milder losses in the distribution area found here, viviparous snakes may also adapt more successfully to the projected climate shifts due to their ability to regulate their body temperature, and also their offspring by moving^40,41^, even over short distances, which could alleviate the distribution losses. Finally, it is worrying from a conservation perspective that the existing PAs of the Atlantic Forest Hotspot are poorly conserving snake species at current time and that they should also fail in protecting the snake diversity in the future. The situation is aggravated by the fact that the PAs are not currently connected to each other to allow the dispersal of the species. Thus, an important step towards a more effective strategy for conservation of biodiversity in Atlantic Forest would be the expansion of the PAs network preferably in connected configurations and that taking into account the effects of future climate change on the species. We call for the need for studies focused on the planning of new PAs, identifying areas that maximize the species occupancy under future warming scenarios, accounting not only for snake richness, but also additional groups of species with greatest conservation need.

## Methods

### Study region

Our analyses were focused on the Atlantic Forest Biodiversity Hotspot^42^, which originally covered around 150 million ha, with heterogeneous environmental conditions provided by a wide range of climatic belts and vegetation formations^43,57^. This biome has a latitudinal range extending into the tropical and subtropical regions, a longitudinal range harbouring differences in forest composition due to a diminishing gradient in rainfall from coast to interior^43^ and an altitudinal range encompassing the elevational limits of the mountain chains of Serra do Mar and Serra da Mantiqueira^100^ (Fig. 7a). Despite high diversity and endemism, currently only 9% of the remaining forest (corresponding to 1% of the original forests), are legally protected^42,43^ (Fig. 7b).

### Species data

We obtained occurrence records for 144 snake species inhabiting the Atlantic Forest from museum records (Species Link, http://splink.cria.org.br/). We only considered species with currently valid scientific names, therefore avoiding known synonyms^101^. We used ArcGIS 10.1 software^102^ to map occurrence records of each species on a regular geographic grid of 10,359 cells with 0.1° (about 10 × 10 km) of spatial resolution covering all of the Atlantic Forest (see Supplementary Fig. S2). Cells corresponding to oceanic islands were excluded from analyses. We removed from analysis the species with recorded occurrence in 4 or less grid cells, since many studies demonstrate that the precision and accuracy of ENMs increase with area of species geographic distribution^103,104^. Thus, the sampling size of the species occurrence data ranged from 5 to 816 occupied cells. We used the “Spatial Join” ArcGIS toolbox to transform species’ spatial occurrences in matrices, matching rows from the join features to the target features based on their relative spatial locations. Then, we combined vector files based on expert knowledge of the species’ ranges into an overall coverage for species distribution modelling. We only considered spatial occurrences by those species where the distribution data intersected at least a grid cell. We also used the “Count Overlapping Polygons” ArcGIS toolbox to obtain the species richness at the spatial resolution assessed, removing all duplicate records from the analyses (i.e., repeated records of a species at a single locality). The 144 species considered for analysis were classified accordingly to their reproductive modes: oviparous (110 species) or viviparous (34 species). It is possible that some areas of the biome have been poorly sampled, resulting in few records of snake in the museums (Fig. S2). Potential distribution modelling is able to deal with species unsatisfactorily sampled; therefore, it can remedy part of this problem.

### Ecological niche modelling, species richness and turnover

Considering that species occurrence patterns are determined at large-spatial scales by responses of organisms to different environmental conditions reflecting the Grinellian component of the ecological niche^105^, we used the ecological niche models (ENMs) to predict the distribution area of snake species in the Atlantic Forest Hotspot. For this analysis, we used the species occurrence matrix and the layers of climatic-environmental variables for building ecological niche models and obtaining a suitability matrix, from which the potential distribution of each species was mapped.

We used the following bioclimatic variables in the modelling process: annual mean temperature, temperature annual range, precipitation of wettest month, precipitation of driest month and precipitation of warmest quarter, which were determined as the most independent by a factorial analysis. These variables were obtained for the present (pre-industrial) and future (mean of simulations for 2080–2100) from CMIP5 – Coupled Models Intercomparison Project Phase 5 (http://cmip-pcmdi.llnl.gov/cmip5/; also available in http://ecoclimate.org)^106^, and downscaled to the resolution of 0.05°. We also used altitude as predictor, which was obtained from Worldclim (www.worldclim.org). We assumed the temporal stationarity (constancy of the altitude values up to 2080–2100) of this variable to perform future predictions. For estimates of future climate, we used the greenhouse gas concentration trajectory corresponding to the Representative Concentration Pathway (RCP) 4.5 that represents a moderated emission scenario within an optimistic context. We used simulations provided by four Atmosphere-Ocean General Circulation Models (AOGCMs), obtained from Coupled Model Intercomparison Project – Phase 5 (CMIP5): Community Climate System Model (CCSM), Centre National de Recherches Météorologiques (CNRM), Model for Interdisciplinary Research on Climate (MIROC) and Meteorological Research Institute (MRI). Original data resolution varied from 1° to 2.8° (in longitude and latitude) and both current and future climate variables were rescaled to fit our grid resolution.

We used four conceptually and statistically different ENMs based on presence data (i.e., only occurrences of snakes species are known, absences are unknown). The models included in the analyses were Bioclim – BIOC^107^ based on bioclimatic envelope logic, Gower Distance – GOWD^108^ based on environmental distance approach, Maximum Entropy – MAXE^109^ based on machine learning technique and Ecological Niche Factor Analysis – ENFA^110^ based on multivariate analysis. Given the particularities of each model, different predictions are provided, generating estimates of uncertainty about which model is more appropriate to represent the geographical distribution of species^111^. To overcome this uncertainty and minimize errors, we employed the ensemble forecasting approach, which provides a consensus of multiple models^89^. The main idea of ensemble forecasting is that different sources of errors will affect each niche model in different ways and, by obtaining a consensus result of these models, errors will tend to cancel each other out and produce a trustworthy and more conservative solution^86^. Assuming that the consensus model (CONS) reduces uncertainty and error associated with alternative ENMs, only the range sizes from the CONS model were interpreted. To obtain the CONS model we first adjust for each species the individual models as follows.

For all individual models, we randomly partitioned presence (and pseudo-absence in the case of Maxent) data of each species into 75% for calibration (or training) and 25% for evaluation (or test); repeating this process 50 times by cross-validation. For each ENM, we converted the continuous predictions of suitability into a binary vector of 1/0 (presence and absence in each cell), finding the threshold that maximizes sensitivity and specificity values in the receiver operating characteristic (ROC) for each model and species. The ROC curve is generated by plotting the fraction of true positives vs. the fraction of false positives at various threshold settings (all the possible values between 0 and 1). Thus, the decision threshold for each model and species corresponded to the value maximizing true positive and minimizing false positive rates. The distribution area of each species was estimated obtaining 800 predictions (4 ENMs × 50 randomizations × 4 AOGCMs) for each species and time period of climatic conditions (present and future). This allowed us to generate a frequency of projections in the ensemble. Then, we generated the frequency of projections weighted by the TSS statistics for the present and future (i.e. the best models according to this metric have more weight in our consensus projections). The TSS range from −1 to +1, where values equal to +1 is a perfect prediction and values equal to or less than zero is a prediction no better than random^112^. The CONS was obtained by considering the species present only in cells where at least 50% of models retained in the ensemble (the best models according to TSS metric) pointed out the species as present. The CONS was done for each AOGCM and time period (present and 2080). Thus, the final maps of richness and turnover for present and future were obtained by averaging richness values projected by CONS for each grid cell in each different AOGCM. All models were run using the computational platform Bioensembles^111^ and maps were done using the software SAM v.4.0^113^.

To determine the species richness patterns of Atlantic Forest snakes, we employed the modelling strategy at the community level of “predict first, assemble later”^114^, which the ranges of individual species are modelled one at a time as a function of environmental predictors and then overlapped for obtaining the species richness value in each grid cell. A principal components analysis – PCA^115^ was used to compare species richness patterns derived from alternative ENMs and their consensus. This analysis allowed us i) to evaluate the degree to which different ENMs converge in estimates of regional species richness and ii) to determine which model reflects the main direction of variation among richness maps^85^. In our study, only the results of the model reflecting the main direction of variation among suitability maps were interpreted. Finally, we calculated species turnover between current and future species distributions in each cell according to formula (G + L/S)/S + G, where “G” was the number of species gained, “L” the number of species lost and “S” is the contemporary species richness found in the cell^116^.

### Conservation status of species facing the climate change

Genuine assessments of the conservation status of species should be based on detailed evaluation under all of the five IUCN Red List criteria (A–E criteria^117^); however, the appropriate rating may be established if any one of the criteria is taken into account^17,117^. As detailed and reliable population-level data are generally lacking for many taxa, especially for tropical species as is the case of the Atlantic Forest snakes, it is not possible to meet the demands of all IUCN criteria. In this sense, the distribution area, the key parameter of B criterion, has been used to provide analyses on the conservation status of data-poor species^118–120^. Thus, from the individual range of each species in current and future times, it was possible to estimate the conservation status of snake species of the Atlantic Forest at the end of this century, according to the Sub criterion B1 (extent of occurrence – EOO). Thus, based on distribution data resulting from future climate shifts, we proposed the following threat categories in a regional context: 1. Extinct (EX): occurrence = 0 km^2^; 2. Critically Endangered (CR): occurrence < 100 km^2^; 3. Endangered (EN): occurrence < 5,000 km^2^; 4. Vulnerable (VU): occurrence < 20,000 km^2^; 5. Nonthreatened (NT): occurrence > 20,000 km^2^. It is important to emphasize that this classification is not intended to provide the current conservation status of the Atlantic Forest snakes, but rather it provides the first indication of a future condition imposed by redistribution of species in response to climate change.

We also evaluated the percentage of range loss for the future based on Maiorano^121^, considering the following categories: I. species whose loss is estimated at <30% for the projected time interval; II. species whose loss is estimated at 30% for the projected time interval; III. species whose loss is estimated at 50% for the projected time interval; IV. species whose loss is estimated at 80% for the projected time interval and V. species whose loss is estimated at 100% for the projected time interval (from present until 2080). It is noteworthy that these approaches only considers the effects of climate change on species distribution, thus providing a synthetic view of the specific threats imposed on the species by the alteration in climatic conditions.

### Protected area effectiveness under climate change

Digital geographic data for all PAs in the Atlantic Forest falling within IUCN categories I-IV^122^ were overlaid onto our grid cells. The 133 PAs comprised 711 cells entirely distributed in the biome. To assess the ability of PAs to effectively protect snake richness within the Atlantic Forest during climate change, we compared the current and future species distribution (given by our CONS model) with the disposition of the PAs. We did this performing two complementary analyses of PAs effectiveness based on null-modelling approach^10,12,79^. The first one indicates how the spatial location of a given protected area determines if it will gain or lose species under different a climate change scenarios^10^. More specifically, we evaluated whether current locations of PAs are better than random allocations in protecting snake species under climate change^10,12^. For this, we generated a null model that considered the original size, form and orientation of protected areas but disregarded intrinsic effects (i.e. latitude and altitude) affecting their suitability in the face of climate change. The null model randomly allocated the protected areas in the Atlantic Forest and obtained species richness in the present and the future based on the projections of species distribution provided by the CONS model. Protected locations were randomized and repeated 1,000 times in order to obtain the average species richness for both climate scenarios. The second analysis demonstrated the effectiveness of the PAs network in protecting snakes by identifying the level of representativeness of species in the PAs at present and future times^79^. For each species, the level of representativeness in the PAs network of Atlantic Forest was calculated as the mean percentage overlap (MPO), which corresponds to the mean percentage of spatial overlap between the units in which the species occurs in the studied area (10,359 grid cells in Atlantic Forest Hotspot) and the protected areas. For that, we firstly obtained the spatial overlap (%) of each cell of the study area with the polygons of PAs. So, we used null models to test if the level of MPO of each species was significantly different (lower or higher) than would be expected by chance, given its range size (e.g., the number of occupied cells in the Atlantic Forest Hotspot). The observed MPO value for a species was compared with MPO values obtained from 1,000 random species with an equivalent range size (i.e., the same number of cells in which the species occurs but extracted randomly from the entire study area). This comparison allowed us to identify if the representativeness of species within PAs given by MPO values is significantly higher and lower than would be expected by chance, considering a significance level of p < 0.05. The two analyzes were developed in the R platform^123^.

## Supplementary information

Supplementary Material
