# Supplementary material for: Climate change will decrease the range size of snake species under negligible protection in the Brazilian Atlantic Forest hotspot
Source: Sci Rep. 2019 Jun 12;9:8523. doi: 10.1038/s41598-019-44732-z (PMC6561978; doi:10.1038/s41598-019-44732-z)
Supplement: Supplementary file 1 — Supplementary Material [file 41598_2019_44732_MOESM1_ESM.doc]

**Title Page**

**Climate change will decrease the range size of snake species under negligible protection in the Brazilian Atlantic Forest hotspot**

Ricardo Lourenço-de-Moraes^1,2*^, Fernando Miranda Lansac-Toha^1^, Leilane Talita Fatoreto Schwind^1^, Rodrigo Leite Arrieira^3^, Rafael Rogério Rosa^1^, Levi Carina Terribile^4^, Priscila Lemes^5^, Thiago Fernando Rangel^6^, José Alexandre Felizola Diniz-Filho^6^, Rogério Pereira Bastos^2,6^, Dayani Bailly^1^

*Correspondence: Ricardo Lourenço-de-Moraes; Programa de Pós-graduação em Ecologia de Ambientes Aquáticos Continentais (PEA), Universidade Estadual de Maringá, Av. Colombo 5790, Bloco G-90, 87020-900, Maringá - PR – Brazil. Email: ricardo_lmoraes@hotmail.com; Phone: +(55)4432381821; Fax: (+55)4432381821

ORCID: 0000-0001-6055-5380

**Supporting Information**

**Climate change will decrease the range size of snake species under negligible protection in the Brazilian Atlantic Forest hotspot**

## Supporting Information captions

Table S1. Values of PCA loadings of the different ecological niche models and consensus for all species in the current time and future (2080).

Table S2. List of studied snake species of Atlantic Forest hotspot with the summarization of the climate change effects on species distribution.

Table S3. Mean percentage of spatial overlap (MPO) between the distribution areas of the viviparous snake species and protected areas of the Atlantic Forest.

Table S3. Mean percentage of spatial overlap (MPO) between the distribution areas of the oviparous snake species and protected areas of the Atlantic Forest.

Figure S1. Loadings of the first two axes of the PCA performed considering the species richness predictions provided by different ENMs.

Figure S2. Original distribution of species used to model of potential distribution.

Table S1. Values of PCA loadings of the different ecological niche models and consensus for all species in the current time and future (2080).

|  | Present  Oviparous | | Present  Viviparous | | Future  Oviparous | | Future  Viviparous | |
| --- | --- | --- | --- | --- | --- | --- | --- | --- |
|  | PC1 | PC2 | PC1 | PC2 | PC1 | PC2 | PC1 | PC2 |
| BIOCLIM | 0.906 | -0.377 | 0.905 | -0.357 | 0.887 | 0.423 | 0.884 | -0.39 |
| ENFA | 0.849 | 0.344 | 0.772 | 0.323 | 0.819 | -0.424 | 0.74 | 0.395 |
| GOWER | 0.845 | -0.501 | 0.848 | -0.484 | 0.881 | 0.432 | 0.865 | -0.437 |
| MAXENT | 0.825 | 0.486 | 0.712 | 0.611 | 0.805 | -0.516 | 0.559 | 0.724 |
| CONS | **0.987** | 0.073 | **0.983** | 0.05 | **0.982** | 0.007 | **0.976** | 0.025 |
| Axes explanation (%) | 0.782 | 0.151 | 0.722 | 0.168 | 0.769 | 0.162 | 0.669 | 0.205 |
| Acummulated explanation (%) | 0.933 | | 0.89 | | 0.931 | | 0.873 | |

**Table S2. List of studied snake species of Atlantic Forest hotspot with the summarization of the climate change effects on species distribution.**

|  |  | **Range size** | | | **Status of threat** | | |
| --- | --- | --- | --- | --- | --- | --- | --- |
| **Reproductive mode/family/species** | **Observed size range** | **Occupied cells in presente (Km^2^)** | **Occupied cells in future (Km^2^)** | **% of lost (-) and gained (+) of cells** | **Brazilian Red List** | **IUCN** | **Projected by 2080** |
| **Oviparous species** |  |  |  |  |  |  |  |
| **Anomalepididae** |  |  |  |  |  |  |  |
| *Liotyphlops beui* | 63 | 213400 | 85100 | -60.1 | Not Evaluated | Least Concern | Least Concern |
| *Liotyphlops ternetzii* | 23 | 133025 | 73200 | -45.0 | Not Evaluated | Not Evaluated | Least Concern |
| **Colubridae** |  |  |  |  |  |  |  |
| *Chironius bicarinatus* | 230 | 237300 | 88850 | -62.6 | Not Evaluated | Not Evaluated | Least Concern |
| *Chironius carinatus* | 54 | 10425 | 10400 | -0.2 | Not Evaluated | Data Deficient | Vulnerable |
| *Chironius exoletus* | 133 | 276300 | 85575 | -69.0 | Not Evaluated | Not Evaluated | Least Concern |
| *Chironius flavolineatus* | 269 | 52575 | 37200 | -29.2 | Not Evaluated | Not Evaluated | Least Concern |
| *Chironius foveatus* | 203 | 59300 | 29875 | -49.6 | Not Evaluated | Not Evaluated | Least Concern |
| *Chironius fuscus* | 96 | 221775 | 64575 | -70.9 | Not Evaluated | Not Evaluated | Least Concern |
| *Chironius laevicollis* | 106 | 258025 | 94125 | -63.5 | Not Evaluated | Not Evaluated | Least Concern |
| *Chironius multiventris* | 66 | 228150 | 74875 | -67.2 | Not Evaluated | Not Evaluated | Least Concern |
| *Chironius quadricarinatus* | 75 | 308850 | 138150 | -55.3 | Not Evaluated | Not Evaluated | Least Concern |
| *Drymarchon corais** | 23 | 229475 | 265700 | 15.7 | Not Evaluated | Least Concern | Least Concern |
| *Drymoluber dichrous* | 15 | 122725 | 37325 | -69.6 | Not Evaluated | Not Evaluated | Least Concern |
| *Leptophis ahaetulla* | 34 | 276300 | 131975 | -52.2 | Not Evaluated | Not Evaluated | Least Concern |
| *Palusophis bifossatus* | 146 | 256900 | 153050 | -40.4 | Not Evaluated | Not Evaluated | Least Concern |
| *Oxybelis aeneus* | 40 | 177300 | 68025 | -61.6 | Not Evaluated | Not Evaluated | Least Concern |
| *Spilotes pullatus* | 183 | 314625 | 134125 | -57.4 | Not Evaluated | Not Evaluated | Least Concern |
| *Spilotes sulphureus* | 39 | 220750 | 112825 | -48.9 | Not Evaluated | Not Evaluated | Least Concern |
| *Tantilla boipiranga* | 7 | 19475 | 11925 | -38.8 | Not Evaluated | Not Evaluated | Vulnerable |
| *Tantilla melanocephala* | 48 | 183075 | 98400 | -46.3 | Not Evaluated | Not Evaluated | Least Concern |
| *Tantilla pallida*** | 112 | 101425 | 54400 | -46.4 | Not Evaluated | Not Evaluated | Least Concern |
| **Dipsadidae** |  |  |  |  |  |  |  |
| *Apostolepis assimilis* | 346 | 92550 | 66000 | -28.7 | Not Evaluated | Not Evaluated | Least Concern |
| *Apostolepis dimidiata* | 15 | 126200 | 32300 | -74.4 | Not Evaluated | Not Evaluated | Least Concern |
| *Apostolepis erythronata*** | 51 | 86150 | 49600 | -42.4 | Not Evaluated | Not Evaluated | Least Concern |
| *Atractus guentheri* | 33 | 250450 | 109400 | -56.3 | Not Evaluated | Not Evaluated | Least Concern |
| *Atractus pantostictus* | 56 | 180300 | 49825 | -72.4 | Not Evaluated | Not Evaluated | Least Concern |
| *Atractus reticulatus* | 74 | 261450 | 95875 | -63.3 | Not Evaluated | Not Evaluated | Least Concern |
| *Atractus serranus* | 15 | 38175 | 7375 | -80.7 | Vulnerable | Not Evaluated | Vulnerable |
| *Atractus trihedrurus* | 26 | 134800 | 57700 | -57.2 | Not Evaluated | Not Evaluated | Least Concern |
| *Atractus zebrinus* | 30 | 189675 | 65925 | -65.2 | Not Evaluated | Not Evaluated | Least Concern |
| *Boiruna maculata* | 14 | 406950 | 77850 | -80.9 | Not Evaluated | Not Evaluated | Least Concern |
| *Boiruna sertaneja* | 5 | 18350 | 1350 | -92.6 | Not Evaluated | Not Evaluated | Endangered |
| *Clelia clelia* | 36 | 44050 | 13225 | -70.0 | Not Evaluated | Not Evaluated | Vulnerable |
| *Clelia hussami* | 6 | 2125 | 0 | -100.0 | Not Evaluated | Data Deficient | Critically Endangered |
| *Clelia plúmbea* | 107 | 269875 | 104050 | -61.4 | Not Evaluated | Not Evaluated | Least Concern |
| *Coronelaps lepidus* | 29 | 235225 | 77950 | -66.9 | Not Evaluated | Not Evaluated | Least Concern |
| *Dipsas albifrons* | 87 | 231175 | 82275 | -64.4 | Not Evaluated | Not Evaluated | Least Concern |
| *Dipsas alternans* | 57 | 202150 | 58550 | -71.0 | Not Evaluated | Not Evaluated | Least Concern |
| *Dipsas bucéfala* | 21 | 147525 | 59175 | -59.9 | Not Evaluated | Not Evaluated | Least Concern |
| *Dipsas catesbyi* | 12 | 77625 | 4100 | -94.7 | Not Evaluated | Least Concern | Endangered |
| *Dipsas indica* | 86 | 286675 | 124675 | -56.5 | Not Evaluated | Not Evaluated | Least Concern |
| *Dipsas indica petersi* | 31 | 214650 | 85325 | -60.2 | Not Evaluated | Not Evaluated | Least Concern |
| *Dipsas mikani* | 204 | 288650 | 110500 | -61.7 | Not Evaluated | Not Evaluated | Least Concern |
| *Dipsas neivai* | 72 | 262250 | 131075 | -50.0 | Not Evaluated | Not Evaluated | Least Concern |
| *Dipsas neuwiedi* | 92 | 88975 | 59775 | -32.8 | Not Evaluated | Not Evaluated | Least Concern |
| *Dipsas turgidus* | 340 | 293025 | 106050 | -63.8 | Not Evaluated | Not Evaluated | Least Concern |
| *Dipsas ventrimaculatus* | 48 | 279425 | 191925 | -31.3 | Not Evaluated | Least Concern | Least Concern |
| *Echinanthera amoena* | 33 | 143350 | 39225 | -72.6 | Not Evaluated | Not Evaluated | Least Concern |
| *Echinanthera cephalostriata* | 69 | 181075 | 60000 | -66.9 | Not Evaluated | Not Evaluated | Least Concern |
| *Echinanthera cyanopleura* | 84 | 291625 | 77850 | -73.3 | Not Evaluated | Not Evaluated | Least Concern |
| *Echinanthera melanostigma* | 45 | 211200 | 68800 | -67.4 | Not Evaluated | Not Evaluated | Least Concern |
| *Echinanthera persimilis* | 55 | 204475 | 72700 | -64.4 | Not Evaluated | Not Evaluated | Least Concern |
| *Echinanthera undulata* | 99 | 170675 | 47175 | -72.4 | Not Evaluated | Least Concern | Least Concern |
| *Elapomorphus quinquelineatus* | 96 | 230250 | 69275 | -69.9 | Not Evaluated | Not Evaluated | Least Concern |
| *Elapomorphus wuchereri* | 13 | 72675 | 2375 | -96.7 | Not Evaluated | Not Evaluated | Endangered |
| *Erythrolamprus aesculapii* | 238 | 288825 | 119675 | -58.6 | Not Evaluated | Least Concern | Least Concern |
| *Erythrolamprus almadensis* | 43 | 260200 | 164975 | -36.6 | Not Evaluated | Not Evaluated | Least Concern |
| *Erythrolamprus amarali* | 49 | 155525 | 38050 | -75.5 | Not Evaluated | Not Evaluated | Least Concern |
| *Erythrolamprus atraventer* | 12 | 84725 | 14475 | -82.9 | Not Evaluated | Not Evaluated | Vulnerable |
| *Erythrolamprus cobellus* | 11 | 69850 | 5325 | -92.4 | Not Evaluated | Not Evaluated | Vulnerable |
| *Erythrolamprus miliaris* | 223 | 314550 | 110275 | -64.9 | Not Evaluated | Not Evaluated | Least Concern |
| *Erythrolamprus poecilogyrus* | 203 | 319400 | 198950 | -37.7 | Not Evaluated | Not Evaluated | Least Concern |
| *Erythrolamprus reginae* | 31 | 268550 | 176200 | -34.4 | Not Evaluated | Not Evaluated | Least Concern |
| *Erythrolamprus typhlus* | 80 | 164025 | 64400 | -60.7 | Not Evaluated | Not Evaluated | Least Concern |
| *Erythrolamprus viridis* | 7 | 140150 | 104000 | -25.8 | Not Evaluated | Least Concern | Least Concern |
| *Helicops angulatus* | 29 | 73150 | 39925 | -45.4 | Not Evaluated | Not Evaluated | Least Concern |
| *Hydrodynastes gigas* | 94 | 186950 | 35400 | -81.1 | Not Evaluated | Not Evaluated | Least Concern |
| *Imantodes cenchoa* | 103 | 298750 | 132725 | -55.6 | Not Evaluated | Not Evaluated | Least Concern |
| *Leptodeira annulata* | 57 | 106450 | 24650 | -76.8 | Not Evaluated | Least Concern | Least Concern |
| *Mussurana montana* | 16 | 169975 | 63675 | -62.5 | Not Evaluated | Not Evaluated | Least Concern |
| *Oxyrhopus clathratus* | 198 | 260950 | 76050 | -70.9 | Not Evaluated | Not Evaluated | Least Concern |
| *Oxyrhopus formosus* | 5 | 35750 | 6300 | -82.4 | Not Evaluated | Not Evaluated | Vulnerable |
| *Oxyrhopus guibei* | 206 | 296575 | 138075 | -53.4 | Not Evaluated | Not Evaluated | Least Concern |
| *Oxyrhopus petolarius* | 64 | 202650 | 65000 | -67.9 | Not Evaluated | Not Evaluated | Least Concern |
| *Oxyrhopus rhombifer* | 115 | 383550 | 177475 | -53.7 | Not Evaluated | Not Evaluated | Least Concern |
| *Oxyrhopus trigeminus* | 9 | 176575 | 67650 | -61.7 | Not Evaluated | Not Evaluated | Least Concern |
| *Phalotris lemniscatus* | 15 | 35750 | 0 | -100.0 | Not Evaluated | Least Concern | Extinct |
| *Phalotris mertensi* | 14 | 96850 | 14900 | -84.6 | Not Evaluated | Not Evaluated | Vulnerable |
| *Philodryas aestivus* | 95 | 288575 | 98125 | -66.0 | Not Evaluated | Not Evaluated | Least Concern |
| *Philodryas arnaldoi* | 9 | 43525 | 4900 | -88.7 | Not Evaluated | Not Evaluated | Endangered |
| *Philodryas olfersi* | 233 | 313475 | 198025 | -36.8 | Not Evaluated | Not Evaluated | Least Concern |
| *Philodryas patagoniensis* | 223 | 275050 | 142300 | -48.3 | Not Evaluated | Not Evaluated | Least Concern |
| *Phimophis guerini* | 14 | 207425 | 152400 | -26.5 | Not Evaluated | Not Evaluated | Least Concern |
| *Pseudoboa haasi* | 46 | 185075 | 68275 | -63.1 | Not Evaluated | Least Concern | Least Concern |
| *Pseudoboa nigra* | 92 | 369275 | 271250 | -26.5 | Not Evaluated | Not Evaluated | Least Concern |
| *Pseudoboa serrana* | 6 | 24800 | 5325 | -78.5 | Not Evaluated | Not Evaluated | Vulnerable |
| *Siphlophis compressus* | 41 | 130575 | 33725 | -74.2 | Not Evaluated | Least Concern | Least Concern |
| *Siphlophis longicaudatus* | 54 | 152775 | 61350 | -59.8 | Not Evaluated | Not Evaluated | Least Concern |
| *Siphlophis pulcher* | 70 | 180550 | 61500 | -65.9 | Not Evaluated | Least Concern | Least Concern |
| *Sordellina punctata* | 42 | 161250 | 27900 | -82.7 | Not Evaluated | Not Evaluated | Least Concern |
| *Taeniophalus affinis* | 129 | 391775 | 201800 | -48.5 | Not Evaluated | Least Concern | Least Concern |
| *Taeniophallus bilineatus* | 23 | 232750 | 63575 | -72.7 | Not Evaluated | Not Evaluated | Least Concern |
| *Taeniophallus occipitalis* | 22 | 267150 | 116950 | -56.2 | Not Evaluated | Not Evaluated | Least Concern |
| *Tropidodryas serra* | 151 | 188600 | 59525 | -68.4 | Not Evaluated | Least Concern | Least Concern |
| *Tropidodryas striaticeps* | 106 | 258225 | 70950 | -72.5 | Not Evaluated | Not Evaluated | Least Concern |
| *Uromacerina ricardinii* | 70 | 191100 | 67050 | -64.9 | Not Evaluated | Not Evaluated | Least Concern |
| *Xenodon dorbignyi* | 7 | 116275 | 18500 | -84.1 | Not Evaluated | Not Evaluated | Vulnerable |
| *Xenodon merremii** | 121 | 420300 | 660200 | 57.1 | Not Evaluated | Not Evaluated | Least Concern |
| *Xenodon neuwiedii* | 212 | 278100 | 74425 | -73.2 | Not Evaluated | Least Concern | Least Concern |
| *Xenodon rabdocephalus* | 33 | 99875 | 15875 | -84.1 | Not Evaluated | Not Evaluated | Vulnerable |
| *Xenopholis scalaris* | 33 | 222000 | 95075 | -57.2 | Not Evaluated | Least Concern | Least Concern |
| **Elapidae** |  |  |  |  |  |  |  |
| *Micrurus altirostris* | 85 | 232450 | 64700 | -72.2 | Not Evaluated | Not Evaluated | Least Concern |
| *Micrurus corallinus* | 290 | 282125 | 101850 | -63.9 | Not Evaluated | Not Evaluated | Least Concern |
| *Micrurus decoratus* | 83 | 176625 | 57350 | -67.5 | Not Evaluated | Not Evaluated | Least Concern |
| *Micrurus frontalis* | 226 | 321975 | 158700 | -50.7 | Not Evaluated | Least Concern | Least Concern |
| *Micrurus ibiboboca* | 22 | 126450 | 70325 | -44.4 | Not Evaluated | Not Evaluated | Least Concern |
| *Micrurus lemniscatus* | 105 | 348725 | 285575 | -18.1 | Not Evaluated | Least Concern | Least Concern |
| **Typhlopidae** |  |  |  |  |  |  |  |
| *Amerotyphlops brongersmianus* | 20 | 292275 | 150875 | -48.4 | Not Evaluated | Not Evaluated | Least Concern |
| **Tropidophidae** |  |  |  |  |  |  |  |
| *Tropidophis paucisquamis* | 34 | 161000 | 59100 | -63.3 | Not Evaluated | Not Evaluated | Least Concern |
| **Viperidae** |  |  |  |  |  |  |  |
| *Lachesis muta* | 43 | 233025 | 136725 | -41.3 | Not Evaluated | Not Evaluated | Least Concern |
|  |  |  |  |  |  |  |  |
| **Viviparous species** |  |  |  |  |  |  |  |
| **Boidae** |  |  |  |  |  |  |  |
| *Boa constrictor amarali* | 105 | 361075 | 187800 | -48.0 | Not Evaluated | Not Evaluated | Least Concern |
| *Boa constrictor constrictor* | 335 | 57200 | 47975 | -16.1 | Not Evaluated | Not Evaluated | Least Concern |
| *Corallus cropanii* | 5 | 2875 | 100 | -96.5 | Vulnerable | Endangered | Endangered |
| *Corallus hortulanus* | 107 | 273950 | 112125 | -59.1 | Not Evaluated | Not Evaluated | Least Concern |
| *Epicrates assisi* | 8 | 135075 | 57600 | -57.4 | Not Evaluated | Not Evaluated | Least Concern |
| *Epicrates cenchria* | 36 | 173875 | 131250 | -24.5 | Not Evaluated | Not Evaluated | Least Concern |
| *Epicrates crassus* | 50 | 317500 | 259125 | -18.4 | Not Evaluated | Not Evaluated | Least Concern |
| *Eunectes murinus* | 23 | 162700 | 155250 | -4.6 | Not Evaluated | Not Evaluated | Least Concern |
| **Dipsadidae** |  |  |  |  |  |  |  |
| *Gomesophis brasiliensis* | 43 | 209550 | 75400 | -64.0 | Not Evaluated | Not Evaluated | Least Concern |
| *Helicops carnicaudus* | 53 | 198900 | 57625 | -71.0 | Not Evaluated | Not Evaluated | Least Concern |
| *Helicops infrataeniatus* | 816 | 193425 | 81225 | -58.0 | Not Evaluated | Not Evaluated | Least Concern |
| *Thamnodynastes hypoconia* | 58 | 201525 | 58325 | -71.1 | Not Evaluated | Not Evaluated | Least Concern |
| *Thamnodynastes longicaudus* | 34 | 152350 | 56925 | -62.6 | Not Evaluated | Not Evaluated | Least Concern |
| *Thamnodynastes nattereri*** | 57 | 223050 | 81875 | -63.3 | Not Evaluated | Not Evaluated | Least Concern |
| *Thamnodynastes pallidus* | 43 | 254775 | 136725 | -46.3 | Not Evaluated | Not Evaluated | Least Concern |
| *Thamnodynastes rutilus* | 23 | 45925 | 700 | -98.5 | Not Evaluated | Least Concern | Endangered |
| *Thamnodynastes strigatus* | 232 | 279075 | 107825 | -61.4 | Not Evaluated | Not Evaluated | Least Concern |
| *Tomodon dorsatus* | 185 | 271000 | 76725 | -71.7 | Not Evaluated | Least Concern | Least Concern |
| *Tomodon ocellatus* | 16 | 40900 | 5700 | -86.1 | Not Evaluated | Not Evaluated | Least Concern |
| **Viperidae** |  |  |  |  |  |  |  |
| *Bothrops alternatus* | 103 | 356300 | 124100 | -65.2 | Not Evaluated | Not Evaluated | Least Concern |
| *Bothrops bilineatus* | 32 | 155750 | 24000 | -84.6 | Not Evaluated | Not Evaluated | Least Concern |
| *Bothrops cotiara* | 46 | 175725 | 41975 | -76.1 | Not Evaluated | Not Evaluated | Least Concern |
| *Bothrops erythromelas* | 287 | 124425 | 63775 | -48.7 | Not Evaluated | Least Concern | Least Concern |
| *Bothrops fonsecai* | 44 | 127825 | 39825 | -68.8 | Not Evaluated | Not Evaluated | Least Concern |
| *Bothrops itapetiningae* | 23 | 215300 | 110475 | -48.7 | Not Evaluated | Least Concern | Least Concern |
| *Bothrops jararaca* | 407 | 346550 | 83200 | -76.0 | Not Evaluated | Not Evaluated | Least Concern |
| *Bothrops jararacussu* | 174 | 292300 | 105175 | -64.0 | Not Evaluated | Least Concern | Least Concern |
| *Bothrops leucurus* | 65 | 146725 | 85975 | -41.4 | Not Evaluated | Not Evaluated | Least Concern |
| *Bothrops lutzi* | 5 | 17600 | 3675 | -79.1 | Not Evaluated | Least Concern | Endangered |
| *Bothrops moojeni* | 46 | 322675 | 208575 | -35.4 | Not Evaluated | Not Evaluated | Least Concern |
| *Bothrops neuwiedii* | 36 | 158950 | 73275 | -53.9 | Not Evaluated | Not Evaluated | Least Concern |
| *Bothrops pauloensis* | 36 | 51550 | 20975 | -59.3 | Not Evaluated | Not Evaluated | Least Concern |
| *Bothrops pirajai* | 6 | 17575 | 600 | -96.6 | Endangered | Vulnerable | Endangered |
| *Crotalus durissus* | 307 | 332800 | 258325 | -22.4 | Not Evaluated | Least Concern | Least Concern |

* Snake species project to be beneficiated by climate change. ** Species may be synonymous (The Reptile Data Base, 2019 <http://www.reptile-database.org/>)

**Table S3.** Mean percentage of spatial overlap (MPO) between the distribution areas of the viviparous snake species and protected areas of the Atlantic Forest Hotspot at present and future (2080) times and results of null models describing the representativeness of species in protected areas: (+) significantly higher than expected by chance, (-) significantly lower than expected by chance and (*) non-significant (*p*<0.05).

| Viviparous | | Present | | | Future | | |
| --- | --- | --- | --- | --- | --- | --- | --- |
| N | Taxa | MPO | MPO  randomised | Representativeness | MPO | MPO randomised | Representativeness |
| sp1 | *Boa constrictor constrictor* | 2.11 | 2.07 | * | 3.10 | 2.07 | + |
| sp2 | *Boa constrictor amarali* | 1.87 | 2.07 | * | 0.50 | 2.08 | - |
| sp3 | *Bothrops alternatus* | 1.08 | 2.08 | - | 0.91 | 2.06 | - |
| sp4 | *Bothrops bilineatus* | 3.22 | 2.06 | + | 5.80 | 2.04 | * |
| sp5 | *Bothrops cotiara* | 1.31 | 2.07 | - | 1.21 | 2.07 | * |
| sp6 | *Bothrops erythromelas* | 1.21 | 2.07 | - | 0.80 | 2.06 | - |
| sp7 | *Bothrops fonsecai* | 2.94 | 2.07 | + | 2.71 | 2.07 | * |
| sp8 | *Bothrops itapetiningae* | 1.37 | 2.06 | - | 0.88 | 2.08 | - |
| sp9 | *Bothrops jararaca* | 2.84 | 2.07 | + | 1.57 | 2.07 | * |
| sp10 | *Bothrops jararacussu* | 3.43 | 2.07 | + | 4.76 | 2.07 | + |
| sp11 | *Bothrops leucurus* | 1.69 | 2.07 | * | 1.71 | 2.08 | * |
| sp12 | *Bothrops lutzi* | 0.03 | 2.02 | * | 8.69 | 2.01 | + |
| sp13 | *Bothrops moojeni* | 0.92 | 2.07 | - | 1.87 | 2.08 | * |
| sp14 | *Bothrops neuwiedii* | 1.34 | 2.07 | - | 0.96 | 2.08 | - |
| sp15 | *Bothrops pauloensis* | 0.98 | 2.05 | - | 2.23 | 2.07 | * |
| sp16 | *Bothrops pirajai* | 1.20 | 2.05 | * | 0.00 | 1.97 | * |
| sp17 | *Corallus cropanii* | 5.97 | 2.03 | * | 40.91 | 2.34 | + |
| sp18 | *Corallus hortulanus* | 3.66 | 2.07 | + | 4.64 | 2.09 | + |
| sp19 | *Crotalus durissus* | 1.45 | 2.07 | - | 1.86 | 2.07 | * |
| sp20 | *Epicrates assisi* | 3.07 | 2.08 | + | 4.21 | 2.07 | + |
| sp21 | *Epicrates cenchria* | 2.38 | 2.07 | * | 2.80 | 2.07 | - |
| sp22 | *Epicrates crassus* | 0.85 | 2.07 | - | 1.45 | 2.07 | - |
| sp23 | *Eunectes murinus* | 0.79 | 2.08 | - | 1.14 | 2.08 | - |
| sp24 | *Gomesophis brasiliensis* | 2.06 | 2.07 | * | 1.46 | 2.10 | * |
| sp25 | *Helicops carnicaudus* | 5.24 | 2.08 | + | 4.46 | 2.08 | + |
| sp26 | *Helicops infrataeniatus* | 1.10 | 2.07 | - | 1.38 | 2.07 | - |
| sp27 | *Thamnodynastes hipoconia* | 4.57 | 2.07 | + | 3.54 | 2.08 | + |
| sp28 | *Thamnodynastes longicaudus* | 6.08 | 2.06 | + | 4.64 | 2.07 | + |
| sp29 | *Thamnodynastes nattereri* | 3.69 | 2.07 | + | 3.67 | 2.06 | + |
| sp30 | *Thamnodynastes pallidus* | 3.22 | 2.07 | + | 4.20 | 2.08 | + |
| sp31 | *Thamnodynastes rutilus* | 1.67 | 2.07 | * | 1.61 | 2.08 | * |
| sp32 | *Thamnodynastes strigatus* | 2.17 | 2.07 | * | 1.27 | 2.07 | - |
| sp33 | *Tomodon dorsatum* | 3.01 | 2.07 | + | 1.94 | 2.08 | * |
| sp34 | *Tomodon ocellatus* | 0.72 | 2.05 | - | 0.64 | 2.05 | * |

**Table S4. Mean percentage of spatial overlap (MPO) between the distribution areas of oviparous snake species and protected areas of the Atlantic Forest Hotspot at present and future (2080) times and results of null models describing the representativeness of the species in protected areas: (+) significantly higher than expected by chance, (-) significantly lower than expected by chance and (*) non-significant (*p*<0.05). EX= species predicted to become extinct over time due to the loss of 100% of their environmental-climatic suitability by 2080.**

| Oviparous | | Present | | | Future | | |
| --- | --- | --- | --- | --- | --- | --- | --- |
| N  Present/Future | Taxa | MPO | MPO randomised | Representativeness | MPO | MPO randomised | Representativeness |
| sp1/sp1 | *Amerotyphlops brongersmianus* | 2.83 | 2.08 | + | 3.20 | 2.07 | + |
| sp2/sp2 | *Apostolepis assimilis* | 2.25 | 2.07 | * | 1.04 | 2.08 | - |
| sp3/sp3 | *Apostolepis dimidiata* | 0.35 | 2.06 | - | 0.94 | 2.05 | * |
| sp4/sp4 | *Apostolepis erythronata* | 1.79 | 2.07 | * | 0.58 | 2.09 | - |
| sp5/sp5 | *Atractus guentheri* | 2.61 | 2.08 | + | 3.86 | 2.06 | + |
| sp6/sp6 | *Atractus pantostictus* | 3.49 | 2.07 | + | 2.43 | 2.07 | * |
| sp7/sp7 | *Atractus reticulatus* | 2.32 | 2.07 | * | 1.54 | 2.07 | * |
| sp8/sp8 | *Atractus serranus* | 5.72 | 2.04 | + | 1.39 | 2.12 | * |
| sp9/sp9 | *Atractus trihedrurus* | 2.80 | 2.06 | + | 2.71 | 2.07 | + |
| sp10/sp10 | *Atractus zebrinus* | 4.75 | 2.07 | + | 2.91 | 2.06 | + |
| sp11/sp11 | *Boiruna maculata* | 2.50 | 2.06 | + | 1.26 | 2.05 | - |
| sp12/sp12 | *Boiruna sertaneja* | 0.00 | 2.10 | - | 2.78 | 2.06 | * |
| sp13/sp13 | *Chironius bicarinatus* | 3.91 | 2.07 | + | 3.08 | 2.08 | + |
| sp14/sp14 | *Chironius carinatus* | 0.00 | 2.07 | - | 0.00 | 2.13 | - |
| sp15/sp15 | *Chironius exoletus* | 4.65 | 2.07 | + | 4.78 | 2.06 | + |
| sp16/sp16 | *Chironius flavolineatus* | 1.61 | 2.07 | * | 0.63 | 2.07 | - |
| sp17/sp17 | *Chironius foveatus* | 2.17 | 2.05 | * | 1.31 | 2.08 | * |
| sp18/sp18 | *Chironius fuscus* | 4.64 | 2.08 | + | 6.23 | 2.06 | + |
| sp19/sp19 | *Chironius laevicollis* | 4.29 | 2.08 | + | 6.35 | 2.06 | + |
| sp20/sp20 | *Chironius multiventris* | 4.79 | 2.07 | + | 4.58 | 2.07 | + |
| sp21/sp21 | *Chironius quadricarinatus* | 2.12 | 2.08 | * | 4.37 | 2.07 | + |
| sp22/sp22 | *Clelia clelia* | 2.47 | 2.09 | * | 1.06 | 2.10 | * |
| sp23/EX | *Clelia hussami* | 0.00 | 2.23 | * | EX | EX | EX |
| sp24/23 | *Clelia plumbea* | 3.66 | 2.06 | + | 4.91 | 2.07 | + |
| sp25/24 | *Coronelaps lepidus* | 3.50 | 2.07 | + | 3.21 | 2.08 | + |
| sp26/25 | *Dipsas albifrons* | 4.50 | 2.06 | + | 4.77 | 2.06 | + |
| sp27/26 | *Dipsas alternans* | 4.99 | 2.06 | + | 3.78 | 2.07 | + |
| sp28/27 | *Dipsas bucephala* | 0.82 | 2.06 | - | 0.54 | 2.07 | - |
| sp29/28 | *Dipsas catesbyi* | 2.17 | 2.07 | * | 11.67 | 2.06 | + |
| sp30/29 | *Dipsas indica* | 3.96 | 2.08 | + | 4.20 | 2.08 | + |
| sp31/30 | *Dipsas indica petersi* | 3.78 | 2.08 | + | 5.69 | 2.07 | + |
| sp32/31 | *Dipsas mikani* | 3.03 | 2.07 | + | 3.47 | 2.06 | + |
| sp33/32 | *Dipsas neivai* | 3.82 | 2.07 | + | 4.53 | 2.07 | + |
| sp34/33 | *Dipsas neuwiedi* | 3.49 | 2.06 | + | 5.27 | 2.06 | + |
| sp35/34 | *Dipsas turgidus* | 2.24 | 2.09 | * | 1.07 | 2.07 | - |
| sp36/35 | *Dipsas ventrimaculatus* | 1.44 | 2.07 | - | 1.07 | 2.06 | - |
| sp37/36 | *Drymarchon corais* | 2.17 | 2.08 | * | 2.54 | 2.07 | + |
| sp38/37 | *Drymoluber dichrous* | 2.01 | 2.06 | * | 2.91 | 2.00 | * |
| sp39/38 | *Echinanthera amoena* | 6.29 | 2.07 | + | 2.80 | 2.08 | * |
| sp40/39 | *Echinanthera cephalostriata* | 5.35 | 2.07 | + | 3.52 | 2.08 | + |
| sp41/40 | *Echinanthera cyanopleura* | 3.34 | 2.07 | + | 2.44 | 2.07 | * |
| sp42/41 | *Echinanthera melanostigma* | 5.08 | 2.07 | + | 4.58 | 2.07 | + |
| sp43/42 | *Echinanthera persimilis* | 4.38 | 2.07 | + | 3.86 | 2.07 | + |
| sp44/43 | *Echinanthera undulata* | 5.65 | 2.07 | + | 2.89 | 2.09 | * |
| sp45/44 | *Elapomorphus quinquelineatus* | 4.51 | 2.06 | + | 3.38 | 2.07 | + |
| sp46/45 | *Elapomorphus wuchereri* | 2.54 | 2.08 | * | 17.48 | 2.06 | + |
| sp47/46 | *Erythrolamprus aesculapii* | 3.74 | 2.07 | + | 3.49 | 2.07 | + |
| sp48/47 | *Erythrolamprus almadensis* | 3.39 | 2.07 | + | 3.62 | 2.07 | + |
| sp49/48 | *Erythrolamprus amarali* | 4.60 | 2.08 | + | 2.34 | 2.10 | * |
| sp50/49 | *Erythrolamprus atraventer* | 3.93 | 2.08 | + | 3.07 | 2.06 | * |
| sp51/50 | *Erythrolamprus cobellus* | 2.60 | 2.08 | * | 16.42 | 2.08 | + |
| sp52/51 | *Erythrolamprus miliaris* | 3.90 | 2.06 | + | 5.47 | 2.08 | + |
| sp53/52 | *Erythrolamprus poecilogyrus* | 3.01 | 2.08 | + | 3.52 | 2.07 | + |
| sp54/53 | *Erythrolamprus reginae* | 3.61 | 2.07 | + | 4.29 | 2.07 | + |
| sp55/54 | *Erythrolamprus typhlus* | 4.95 | 2.08 | + | 2.96 | 2.06 | + |
| sp56/55 | *Erythrolamprus viridis* | 3.03 | 2.07 | + | 3.40 | 2.06 | + |
| sp57/56 | *Helicops angulatus* | 1.36 | 2.08 | * | 0.54 | 2.08 | - |
| sp58/57 | *Hydrodynastes gigas* | 1.16 | 2.07 | - | 2.06 | 2.07 | * |
| sp59/58 | *Imantodes cenchoa* | 4.72 | 2.08 | + | 3.60 | 2.07 | + |
| sp60/59 | *Lachesis muta* | 3.79 | 2.07 | + | 6.42 | 2.07 | + |
| sp61/60 | *Leptodeira annulata* | 0.69 | 2.06 | - | 2.80 | 2.07 | * |
| sp62/61 | *Leptophis ahaetulla* | 2.14 | 2.08 | * | 4.49 | 2.07 | + |
| sp63/62 | *Liotyphlops beui* | 3.77 | 2.08 | + | 2.38 | 2.07 | * |
| sp64/63 | *Liotyphlops ternetzii* | 2.27 | 2.06 | * | 1.36 | 2.06 | - |
| sp65/64 | *Palusophis bifossatus* | 3.40 | 2.07 | + | 3.26 | 2.07 | + |
| sp66/65 | *Micrurus altirostris* | 1.55 | 2.07 | - | 0.91 | 2.07 | - |
| sp67/66 | *Micrurus corallinus* | 4.05 | 2.06 | + | 4.96 | 2.08 | + |
| sp68/67 | *Micrurus decoratus* | 3.47 | 2.09 | + | 2.43 | 2.09 | * |
| sp69/68 | *Micrurus frontalis* | 1.44 | 2.07 | - | 1.71 | 2.07 | * |
| sp70/69 | *Micrurus ibiboboca* | 2.77 | 2.07 | + | 3.45 | 2.07 | + |
| sp71/70 | *Micrurus lemniscatus* | 2.02 | 2.07 | * | 3.00 | 2.07 | + |
| sp72/71 | *Mussurana montana* | 4.71 | 2.08 | + | 3.54 | 2.07 | + |
| sp73/72 | *Oxybelis aeneus* | 2.54 | 2.07 | + | 2.27 | 2.06 | * |
| sp74/73 | *Oxyrhopus clathratus* | 3.24 | 2.08 | + | 3.20 | 2.06 | + |
| sp75/74 | *Oxyrhopus formosus* | 2.26 | 2.06 | * | 13.10 | 2.06 | + |
| sp76/75 | *Oxyrhopus guibei* | 2.19 | 2.07 | * | 2.87 | 2.07 | + |
| sp77/76 | *Oxyrhopus petolarius* | 3.02 | 2.07 | + | 3.02 | 2.05 | + |
| sp78/77 | *Oxyrhopus rhombifer* | 1.91 | 2.06 | * | 1.38 | 2.06 | - |
| sp79/78 | *Oxyrhopus trigeminus* | 1.29 | 2.07 | - | 0.84 | 2.09 | * |
| sp80/EX | *Phalotris lemniscatus* | 1.23 | 2.12 | * | EX | EX | EX |
| sp81/79 | *Phalotris mertensi* | 0.53 | 2.05 | - | 8.16 | 1.99 | * |
| sp82/80 | *Philodryas aestivus* | 2.37 | 2.08 | * | 1.32 | 2.08 | - |
| sp83/81 | *Philodryas arnaldoi* | 1.06 | 2.09 | - | 0.73 | 2.06 | * |
| sp84/82 | *Philodryas olfersi* | 3.07 | 2.07 | + | 2.73 | 2.07 | + |
| sp85/83 | *Philodryas patagoniensis* | 2.99 | 2.07 | + | 2.53 | 2.07 | + |
| sp86/84 | *Phimophis guerini* | 2.41 | 2.09 | * | 1.91 | 2.07 | * |
| sp87/85 | *Pseudoboa haasi* | 2.43 | 2.07 | * | 1.87 | 2.05 | * |
| sp88/86 | *Pseudoboa nigra* | 2.01 | 2.07 | * | 2.79 | 2.07 | + |
| sp89/87 | *Pseudoboa serrana* | 4.75 | 2.02 | + | 1.12 | 2.02 | * |
| sp90/88 | *Siphlophis compressus* | 3.16 | 2.08 | + | 5.35 | 2.10 | + |
| sp91/89 | *Siphlophis longicaudatus* | 5.71 | 2.07 | + | 3.57 | 2.08 | + |
| sp92/90 | *Siphlophis pulcher* | 5.47 | 2.06 | + | 4.51 | 2.07 | + |
| sp93/91 | *Sordelina punctata* | 3.62 | 2.08 | + | 1.64 | 2.05 | * |
| sp94/92 | *Spilotes pullatus* | 3.69 | 2.07 | + | 4.08 | 2.07 | + |
| sp95/93 | *Spilotes sulphureus* | 2.64 | 2.06 | + | 3.45 | 2.09 | + |
| sp96/94 | *Taeniophalus affinis* | 2.40 | 2.07 | + | 2.14 | 2.07 | * |
| sp97/95 | *Taeniophallus bilineatus* | 1.59 | 2.06 | * | 0.03 | 2.05 | - |
| sp98/96 | *Taeniophallus occipitalis* | 3.54 | 2.07 | + | 3.53 | 2.07 | + |
| sp99/97 | *Tantilla boipiranga* | 1.39 | 2.04 | * | 1.14 | 2.10 | * |
| sp100/98 | *Tantilla melanocephala* | 3.30 | 2.07 | + | 1.42 | 2.07 | - |
| sp101/99 | *Tantilla pallida* | 2.15 | 2.09 | * | 0.82 | 2.06 | - |
| sp102/100 | *Tropidodryas serra* | 5.48 | 2.07 | + | 3.80 | 2.07 | + |
| sp103/101 | *Tropidodryas striaticeps* | 4.88 | 2.08 | + | 3.76 | 2.06 | + |
| sp104/102 | *Tropidophis paucisquamis* | 5.67 | 2.07 | + | 4.95 | 2.06 | + |
| sp105/103 | *Uromacerina ricardinii* | 5.25 | 2.06 | + | 3.86 | 2.08 | + |
| sp106/104 | *Xenodon dorbignyi* | 0.65 | 2.06 | - | 1.63 | 1.87 | * |
| sp107/105 | *Xenodon merremii* | 1.82 | 2.07 | * | 2.23 | 2.07 | + |
| sp108/106 | *Xenodon neuwiedii* | 4.36 | 2.07 | + | 3.45 | 2.07 | + |
| sp109/107 | *Xenodon rabdocephalus* | 1.65 | 2.09 | * | 0.26 | 2.07 | - |
| sp110/108 | *Xenopholis scalaris* | 4.23 | 2.07 | + | 4.85 | 2.07 | + |


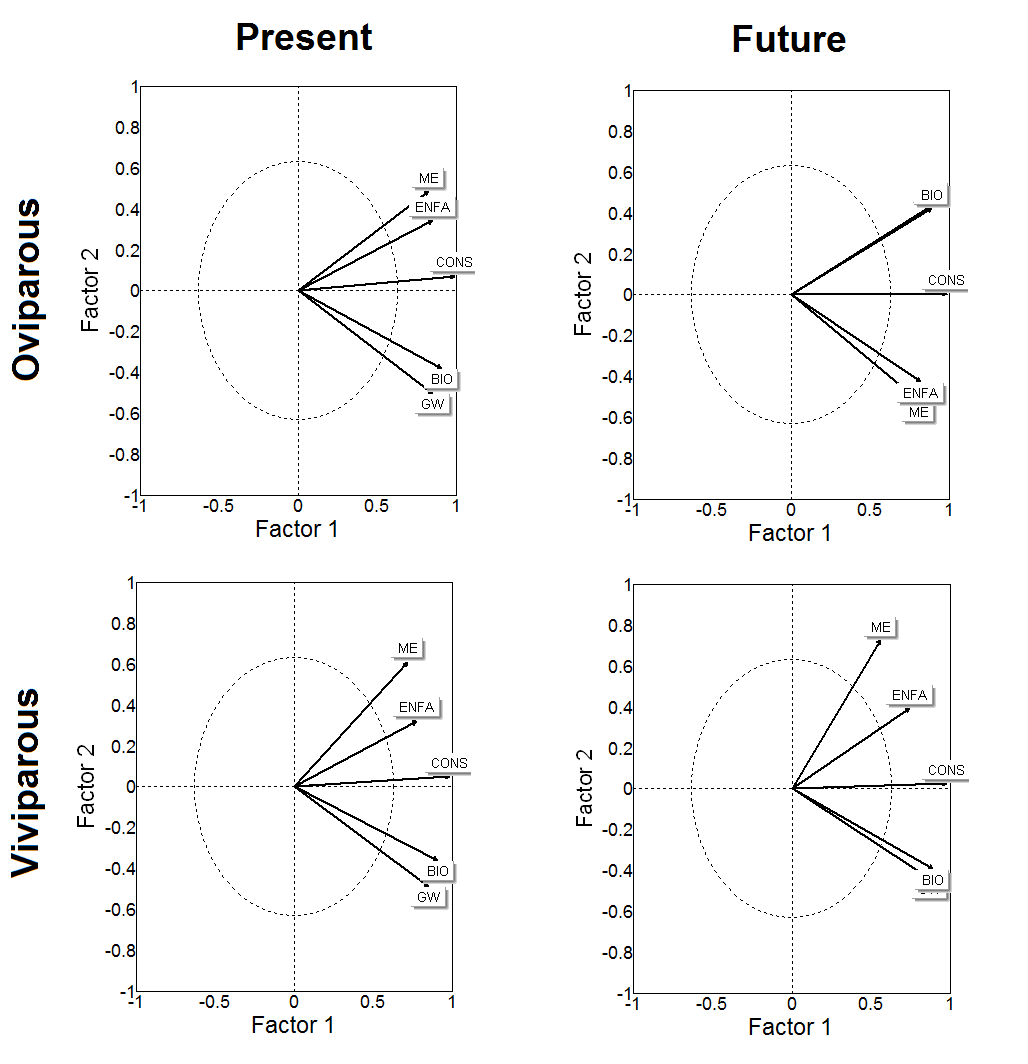


Figure S1. Loadings of the first two axes of the PCA performed considering the species richness predictions provided by different ENMs. Species richness patters forecasted by different ecological niche models (BIO = Bioclim; GOW = Gower distance; MAX = Maximum Entropy; ENF = Ecological Niche Factorial Analysis) for oviparous snakes in the current and future times and for viviparous snakes species in the current and future times.


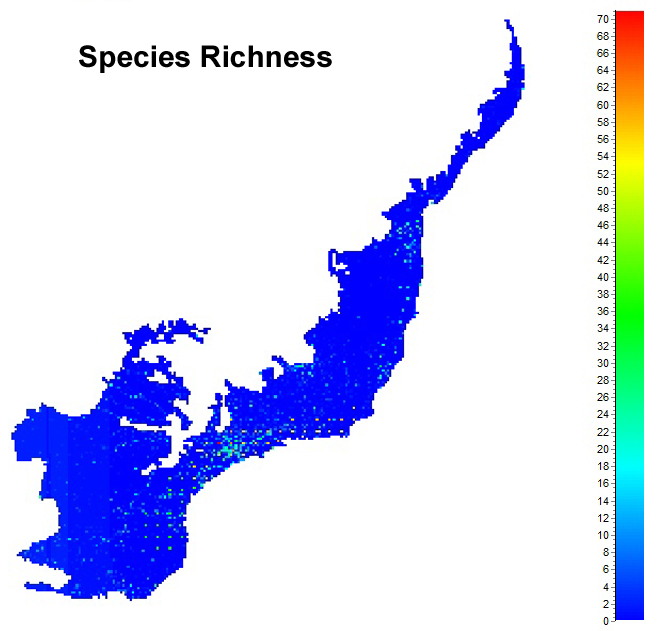


Figure S2. Original distribution of species used to model of potential distribution.
